# Hematemesis as an Initial Presentation of Enteropathy-Associated T-Cell Lymphoma

**DOI:** 10.7759/cureus.16992

**Published:** 2021-08-08

**Authors:** Mehak Bassi, Sonmoon Mohapatra, Parth Sharma, Andrew Korman, C.S. Pitchumoni, Arkady Broder

**Affiliations:** 1 Division of Internal Medicine, Saint Peter’s University Hospital/Rutgers Robert Wood Johnson School of Medicine, New Brunswick, USA; 2 Division of Gastroenterology and Hepatology, Saint Peter’s University Hospital/Rutgers Robert Wood Johnson School of Medicine, New Brunswick, USA; 3 Department of Internal Medicine, All India Institute of Medical Sciences, New Delhi, New Delhi, IND

**Keywords:** non-responsive celiac disease, gluten-free diet, enteropathy associated t-cell lymphoma, intestinal t-cell lymphoma, extranodal lymphomas

## Abstract

Enteropathy-associated T-cell lymphoma (EATL) is a tumor of intraepithelial T-lymphocytes arising in the small intestine. Based on the genetic profile, immunohistochemistry, and histology, EATL is divided into two subtypes. EATL type I occurs in individuals with celiac disease (CD) while EATL type II is a sporadic form that occurs in individuals without CD. Intensive chemotherapy and surgery are the mainstay treatment. However, despite the currently available treatment options, the five-year survival rate is only 9%. EATL presents as abdominal pain, nausea, or slow gastrointestinal bleeding. Severe bleeding leading to hemodynamic instability is rarely known in EATL. Therefore, we present a unique case of EATL who presented with acute and severe gastrointestinal bleeding with no prior history of CD.

## Introduction

Enteropathy-associated T-cell lymphoma (EATL) is a T-cell lymphoma of the small intestine. It usually presents as abdominal pain and chronic gastrointestinal bleeding. However, EATL presenting as massive upper gastrointestinal bleeding is rare. The diagnosis of EATL is challenging and the clinical outcome of the patients is dismal. We present an illustrative report of an EATL case who presented with massive gastrointestinal bleeding with no previous history of CD.

## Case presentation

A 73-year-old Caucasian gentleman presented to the emergency department with massive hematemesis and melena. He also reported intermittent episodes of abdominal pain for three months. After adequate fluid resuscitation, he underwent upper endoscopy and colonoscopy which failed to localize the source of bleeding. Eventually, the bleeding stopped on its own and he was transferred to our hospital for further investigations. He did not report any prior similar episodes. On presentation to our hospital, his vital signs were normal. Physical examination revealed tenderness in the mid-abdomen region.

**Figure 1:**
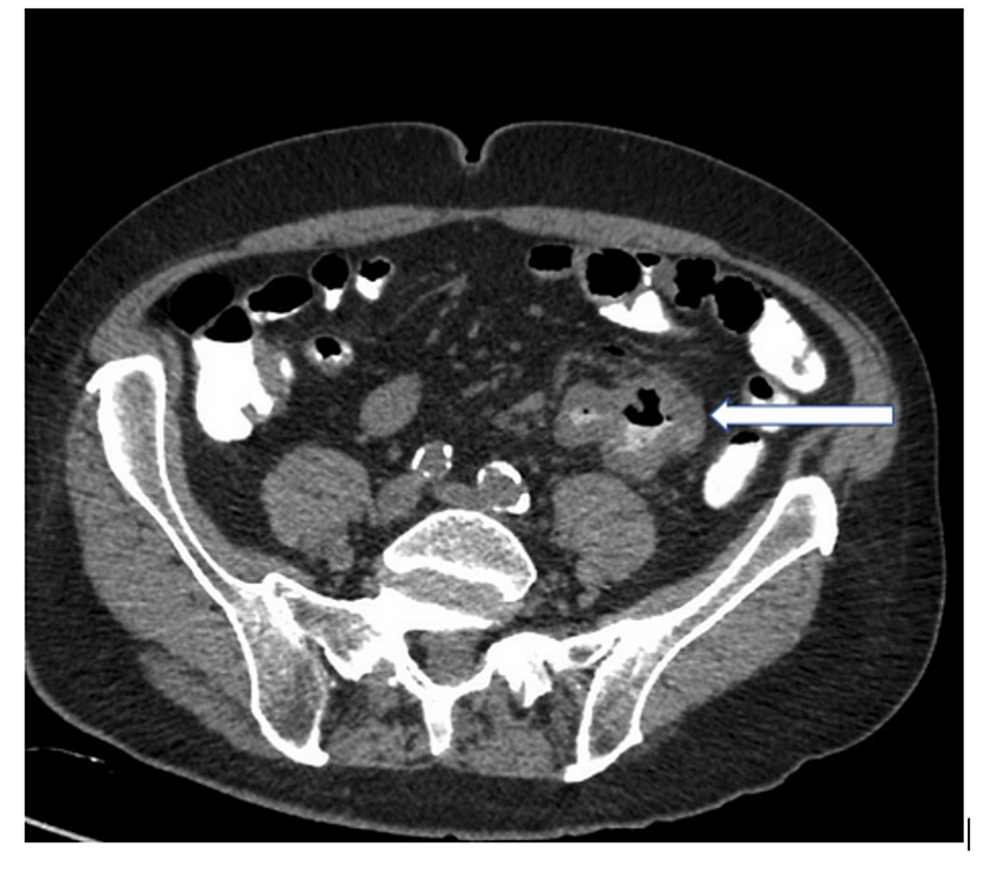
A 5.2 cm × 4.6 cm irregular mass involving the jejunum

Laboratory investigations demonstrated anemia with hemoglobin of 9.4 g/dl, platelet count of 95 × 10^3^/mm^3^, total bilirubin of 1.2 mg/dl, and albumin of 2.2 g/dl. An upper and lower endoscopic procedure performed again showed no stigmata of bleeding. Because of the persistent abdominal pain, he underwent computed tomography (CT) of the abdomen and pelvis with contrast which showed a 5.2 × 4.6 cm^2^ irregular mass involving the jejunum, with wall thickening about 1 cm with adjacent mesentery involving 1.2 cm rounded lymph nodes (Figure 1). Several bilateral lower lobe predominant pulmonary nodules were demonstrated in the CT scan.

A small bowel enteroscopy was performed which demonstrated a 10 cm, circumferential, polypoid, sessile infiltrative, and fungating mass in the mid-jejunum. Biopsy from the jejunal mass showed atypical T-cell infiltrates consistent with malignant T-cell lymphoma. It contained 10 % of the lymphocytes, the majority expressed CD2 (82%) and less CD3(51%), 9% of cells coexpress CD4/CD8 phenotype, which was consistent with enteropathy associated with T-cell lymphoma.

**Figure 2:**
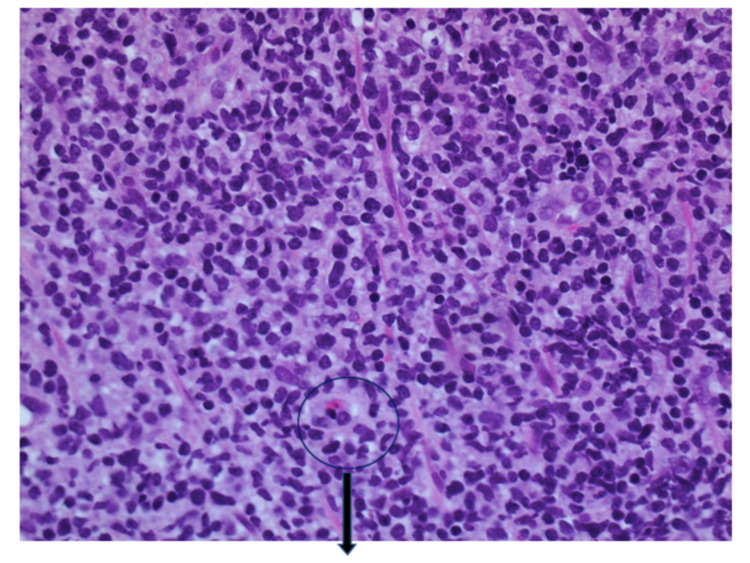
Biopsy of the Jejunal mass: the majority of the lymphocytes expressed CD2

Immunostains were performed which revealed a population of T cells that are positive for CD3 and BCL2. Ki 67 showed a high proliferation rate (Figure 2).

**Figure 3:**
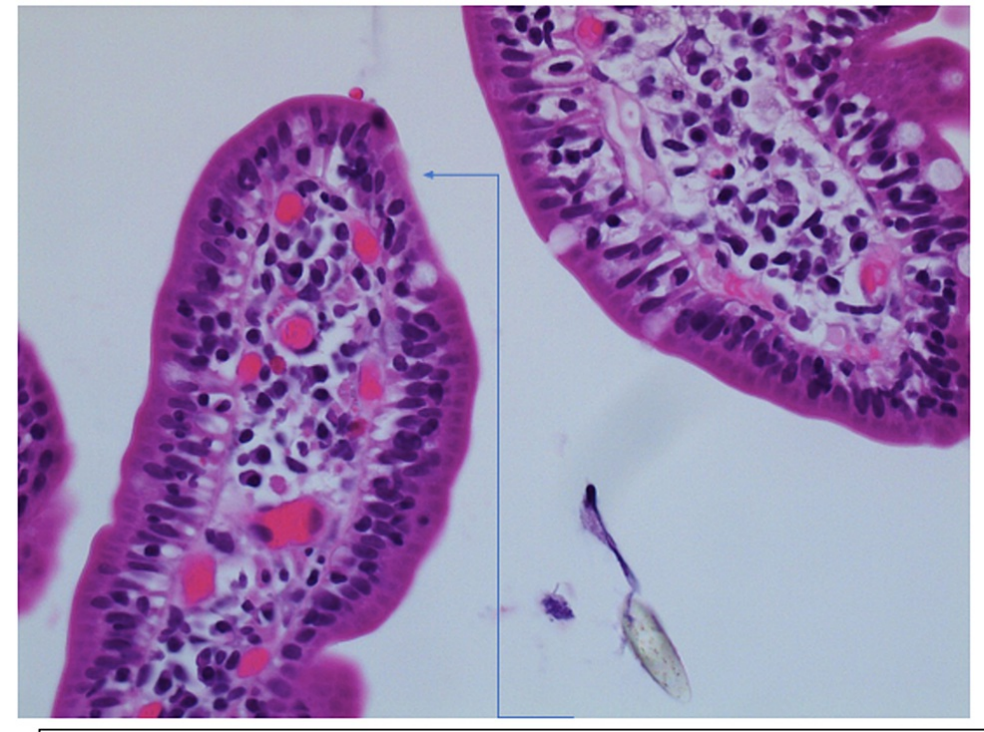
Biopsy of the duodenal bulb: duodenal mucosa with focal epithelial infiltration of the lymphocytes

Flow cytometry showed T-cell positive for CD2, CD3, and CD7, with significantly decreased expression of CD5 and negative for CD4 and CD8. This pattern of expression is in support of T-cell lymphoma. The biopsy of the duodenal bulb demonstrated duodenal mucosa with focal epithelial infiltration of lymphocytes and focal villous blunting suggestive of CD (Figure 3).

**Figure 4:**
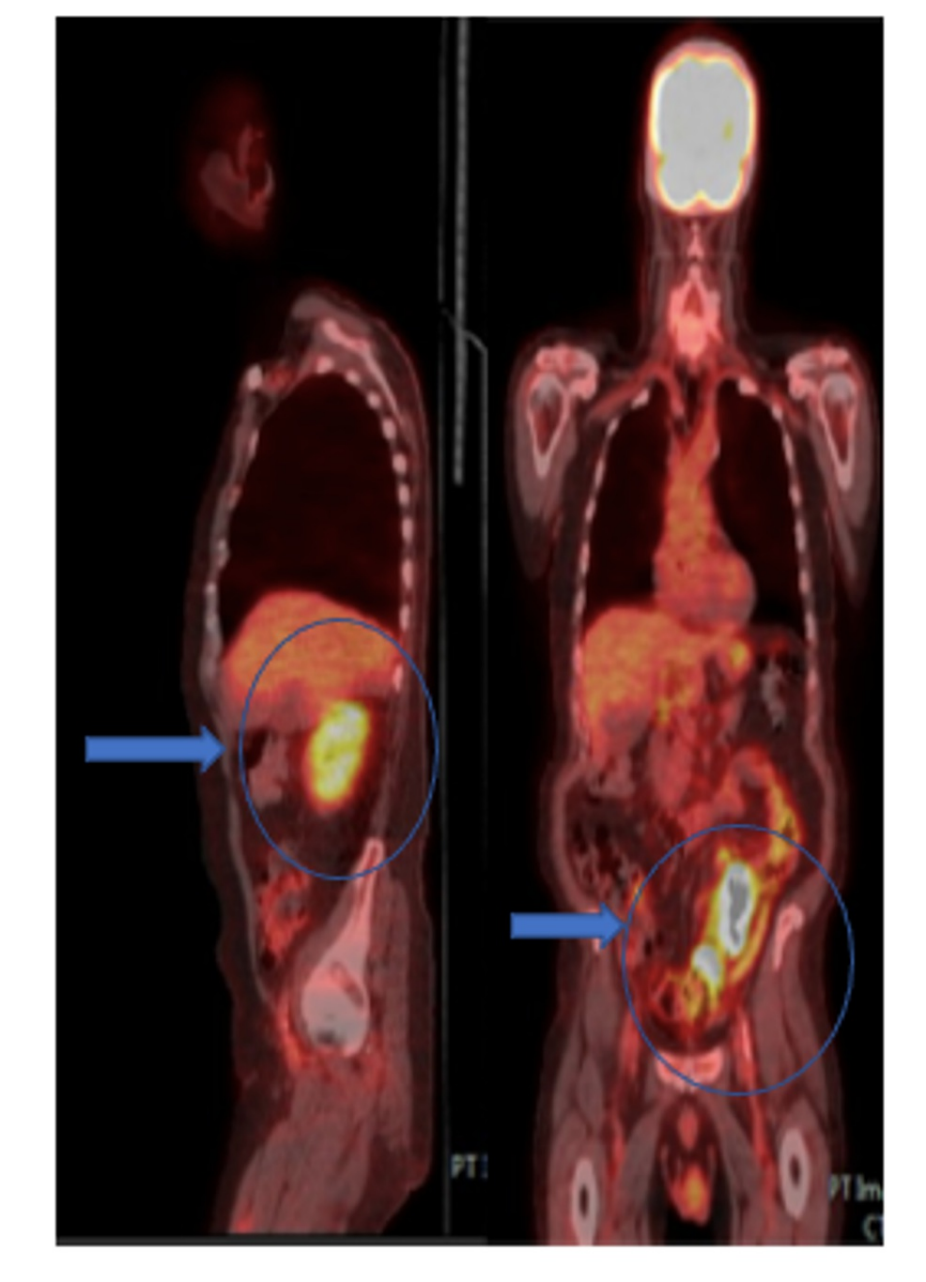
A saggital and coronal view of the PET scan demonstrating uptake in the jejunum with surrounding mesenteric lymph nodes

A PET scan showed a 15 cm markedly thickened jejunal wall. Increased uptake was seen in mesenteric lymph nodes, compatible with lymph node metastases (Figure 4).

Although nonspecific, given the hypermetabolic nature of pulmonary nodules, they were considered malignant based on the clinical context. This high-grade tumor with metastasis to lymph nodes and lungs, pushed the multidisciplinary team to pursue a palliative course of action. The patient ultimately chose to be on hospice care.

## Discussion

EATL is a rare tumor with a slightly higher male predominance and presents at a median age of 60. EATL is peripheral or extranodal T-cell lymphoma arising in the small intestine and is commonly associated with CD [1]. It accounts for 4.2% of all peripheral T-cell lymphomas, which is more common in Europeans due to the increased incidence of celiac disease [2]. In the US, the incidence rate was 0.111 per 1,000,000, with a higher incidence in Asians/Pacific Islanders [3,4]. The frequent site of involvement is observed in the jejunum, followed by the proximal ileum, colon, stomach, liver, and spleen [5,6]. It usually presents as abdominal pain, diarrhea, and weight loss. Small bowel obstruction and perforation are also frequently seen and may be the presenting symptom [7]. The tumor presents in forms of strictures, multifocal nodules, ulcers, and maybe even large masses, infiltrating mesenteric lymph nodes [8-10]. Slow gastrointestinal bleeding or occult bleeding has been reported in EATL [11]. Although, there are only a few case reports in the literature of EATL manifesting as sudden gastrointestinal bleeding [12,13]. Therefore, our case highlights a unique presentation of EATL, which presented as overt life-threatening bleeding.

The World Health Organization (WHO) describes two variants of EATL. Around 80-90% of the cases are type I EATL and type II EATL, a monomorphic variant of the disease [14]. Type 1 (classical) EATL is associated with the CD, with several of them having the CD-associated HLA-DQA1*0501, DBQ*0201 genotypes, and therefore, mostly seen in Northern Europe [15,16]. More than half of the patients with refractory celiac disease (RCD) are at increased risk of developing an EATL within five years. Adherence to a gluten-free diet has been shown to have a protective role against EATL type 1 lymphoma development [16,17]. EATL type 1 has CD3+, CD4-, CD8-, CD5-, CD7+, CD103+, CD56-, TCR-beta +/-cells, which mostly express CD30. In our case, since the histology was positive for CD3+ and negative for CD30, our patient had EATL type 1 lymphoma. The duodenal biopsy of our patient showed focal villous blunting with focal epithelial infiltration of lymphocytes which was consistent with celiac disease.

Type 2 EATL is most common in Asia, presents as a sporadic form with no prior history of CD [9]. The tumor cells express CD8+, CD4−, CD3+, and CD56+ [18,19]. A thorough diagnosis of EATL usually involves double-balloon enteroscopy (DBE) or video capsule enteroscopy (VCE) to obtain histological samples. Video capsule endoscopy (VCE) demonstrates the location and extent of complications like small intestinal lesions and GI bleeding [20,21]. For staging of lymphoma and the presence of extranodal involvement, 18F-fluorodeoxyglucose positron emission tomography scan (18F-FDG-PET scan) and CT should be pursued [21,22]. PET scan is a more sensitive modality than CT for identifying EATL [23].

Non-compliance to a gluten-free diet, delayed diagnoses, age of diagnoses, and genomic homozygosity for HLA- DQ2 are identifiable as risk factors for malignant complications [6]. Combined modality therapy includes surgery with the possibility of subsequent anthracycline-based chemotherapy. Depending on tumor location, surgery is often utilized as a diagnostic and therapeutic modality [15]. The response rate of Anthracycline plus the CHOP regimen ranges between 42% and 58%, while the five-year overall survival is around 20% [6,12]. Alemtuzumab, a monoclonal antibody targeting CD52 on cancerous and healthy T cells, is used with chemotherapy with initially promising results for the remission of diseases [22]. Autologous stem cell transplantation is proven beneficial in selected patients with overall survival of 59% at four years [23]. 

Many studies have emphasized the importance of a gluten-free diet to avoid progression from celiac disease to T-cell lymphoma [24]. Developing newer diagnostic models, early referral to specialized centers, and early surgical intervention is warranted to improve patients' survival. Counseling patients with celiac disease regarding adherence to a gluten-free diet is of utmost importance. In addition to the literature, our case presenting as sudden gastrointestinal bleeding demonstrates an unusual presentation of EATL.

## Conclusions

Enteropathy-associated T-cell lymphoma is an aggressive lymphoma of the small intestine. EATL usually presents as abdominal pain, vomiting, or gastrointestinal perforation. It is known to cause anemia by occult bleeding. However, our case presented with sudden and massive gastrointestinal bleeding as the initial manifestation. The prognosis of this disease is dismal. In this case, we review the current literature and raise awareness of an uncommon presentation of a rare tumor.
